# Systems biology of autophagy in leishmanial infection and its diverse role in precision medicine

**DOI:** 10.3389/fmolb.2023.1113249

**Published:** 2023-04-21

**Authors:** Vrushali Guhe, Prajakta Ingale, Anil Tambekar, Shailza Singh

**Affiliations:** National Centre for Cell Science, NCCS Complex, Ganeshkhind, SPPU Campus, Pune, India

**Keywords:** autophagy, leishmaniasis, lipid droplets, miRNA, systems biology, artificial intelligence, anti-leishmanial drugs, computational biology

## Abstract

Autophagy is a contentious issue in leishmaniasis and is emerging as a promising therapeutic regimen. Published research on the impact of autophagic regulation on *Leishmania* survival is inconclusive, despite numerous pieces of evidence that *Leishmania* spp. triggers autophagy in a variety of cell types. The mechanistic approach is poorly understood in the *Leishmania* parasite as autophagy is significant in both *Leishmania* and the host. Herein, this review discusses the autophagy proteins that are being investigated as potential therapeutic targets, the connection between autophagy and lipid metabolism, and microRNAs that regulate autophagy and lipid metabolism. It also highlights the use of systems biology to develop novel autophagy-dependent therapeutics for leishmaniasis by utilizing artificial intelligence (AI), machine learning (ML), mathematical modeling, network analysis, and other computational methods. Additionally, we have shown many databases for autophagy and metabolism in *Leishmania* parasites that suggest potential therapeutic targets for intricate signaling in the autophagy system. In a nutshell, the detailed understanding of the dynamics of autophagy in conjunction with lipids and miRNAs unfolds larger dimensions for future research.

## Graphical Abstract

**Figure:**
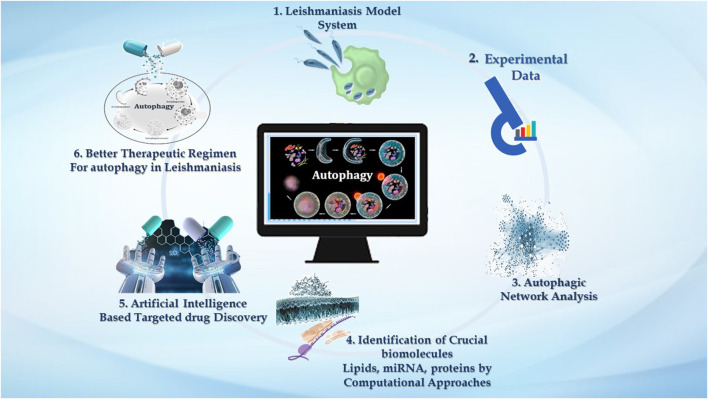
We demonstrate the significance of computational approaches in studying autophagy in leishmaniasis. 1. Macrophages are the major phagocytic cells where these *Leishmania* parasites reside, and via invading immune signaling, it establishes successful infection. 2. Very large data reported in the literature accentuating experimental evidence related to Leishmaniasis embrace host autophagy and parasite autophagy, and the impact of host autophagy on parasite survival. 3. Experimental knowledge can be used to analyze the intricate autophagic network. Numerous online databases contribute to providing insight into the autophagic process in disease. 4. Systems approaches such as mathematical model reconstruction and network analysis help emphasize the autophagy process—interconnected with three pivotal biomolecules, i.e., lipids, proteins, and miRNAs. 5. Computational network analysis is decisive for target identification, which can further be utilized by AI-based tools to screen new drugs for leishmaniasis that impacts autophagy. 6. Drug targets identified by AI-based techniques emphasized targeting autophagy as an improved treatment regimen to combat leishmaniasis.

## 1 Background

The obligate intracellular parasitic protozoa of genus *Leishmania* are an etiological agent of a complex vector-borne zoonotic disease named leishmaniasis. It gets transmitted by more than 20 species of sand fly vectors of the genera *Phlebotomus* and *Lutzomyia* (Djune Yemeli et al., 2021). According to the 2010 WHO expert committee report, different clinical manifestations that occurred in the Old World are classified into three primary forms: 1) visceral leishmaniasis (VL), also known as kala-azar, caused by *L. donovani* and *L. infantum*; 2) cutaneous leishmaniasis (CL), most frequently caused by *L. tropica*, *L. major*, and *L. aethiopica*; and 3) mucocutaneous leishmaniasis (MCL) (can be caused by any species). 4) diffused cutaneous leishmaniasis (caused by *L. aethiopica*) and 5) post kala-azar dermal leishmaniasis (present in all areas with *L. donovani*). While CL is the most common form of the disease, VL is the most serious and is almost always fatal if untreated (Colmenares et al., 2002).

### 1.1 Epidemiology of leishmaniasis

This group of neglected diseases occurs in 98 countries with 12 million cases at risk and 20,000–40,000 deaths per year, according to the World Health Organization (WHO) report (https://www. who. int/news-room/fact-sheets/detail/leishmaniasis). The annual incidence of new cases varies from 0.2 to 0.4 million and 0.7–1.2 million cases per year for visceral and cutaneous leishmaniasis, respectively (Sabzevari et al., 2021). Leishmaniasis is prevalent in Africa, Latin America, Asia, the Mediterranean basin, and the Middle East. Although the cutaneous form (CL) of the disease accounts for more than 50% of new cases of leishmaniasis, 90% of CL cases are found in South America, the Middle East, and Afghanistan. Five nations that account for the majority of VL cases are *viz.*, India, Bangladesh, Ethiopia, Sudan, and Brazil (Modabber, 2010). Although humans are the only known hosts for *L. donovani*, the disease is mostly zoonotic in origin, with canine species serving as the primary animal reservoir. Within a few years after surviving VL, between 20% and 60% of *L. donovani*-infected patients would acquire post kala-azar dermal leishmaniasis (PKDL). Patients with PKDL are believed to be a major source of parasites for new infections because of the large number of parasites identified in their skin (Kedzierski & Evans, 2014).

### 1.2 Life cycle of *Leishmania* parasite

**FIGURE 1:**
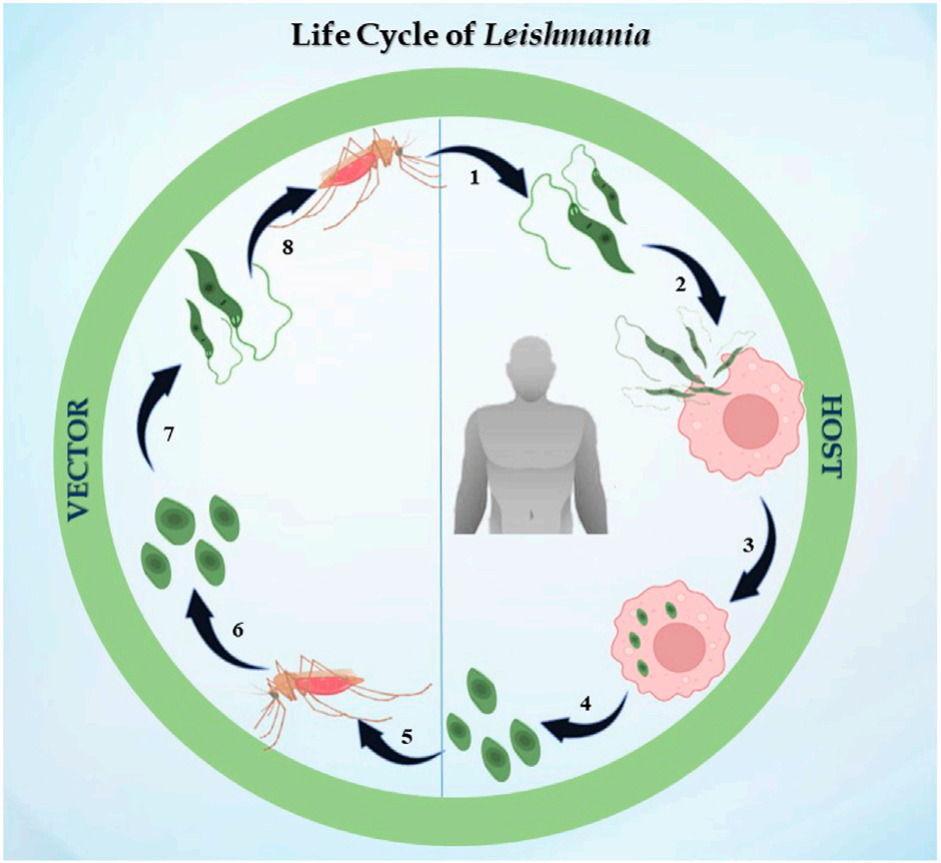
Life cycle of *Leishmania*: the figure represents the digenetic life cycle of 1. promastigotes injected in the host by sandfly, 2. promastigotes engulfed by macrophages of the host, 3. promastigotes transformed into amastigotes inside macrophages, 4. amastigotes transmitted to other healthy macrophages, 5. an infected sandfly taking its next blood meal, 6. amastigotes transforming into the promastigote stage in the midgut, 7. dividing into the midgut and migrating to the proboscis, and 8. the life cycle continues.

The life cycle of *Leishmania* is digenetic, i.e., it alternates between two hosts, i.e., mammalian and insect (Figure 1). In the case of leishmaniasis, the insect vector is the sandfly belonging to the *Phlebotomus* genus in Old World *Leishmania* species and *Lutzomyia* genus for New World *Leishmania* species. After consuming blood from an infected host, the sandfly becomes infected for the first time. The parasite differentiates once it is inside the sandfly, developing into procyclic promastigotes. Promastigotes are flagellated and motile forms of the parasite (Killick-Kendrick, 1974). Their elongated, ovoid body is 1.5–3.5 mm wide and 15–20 mm long. The parasite binds to the sandfly’s gut because the length of its flagella varies from 15 to 28 μm. The procyclic forms split in the midgut of the sandfly, where they produce nectomonad promastigotes that do not divide. These nectomonad promastigotes migrate from the abdominal midgut to the anterior midgut and then transform into leptomonad promastigotes. In due course, the leptomonad promastigotes finally differentiate into metacyclic promastigotes and travel to the sandfly’s proboscis, where they are prepared for transmission to a mammalian host (Harizanov and Kaftandjiev Iskren, 2014). Metacyclic promastigotes are introduced into the host through bite, and upon adhering to the plasma membrane, these promastigotes initiate a phagocytic process. In this manner, the promastigotes enter the macrophage and infect the parasitophorous vacuole. The promastigotes develop into ovoid amastigotes, which have a diameter of 2–4 μm. Inside the parasitophorous vacuole, the amastigotes grow and multiply to the point where the macrophage ruptures, releasing all of the mature amastigotes. The cycle continues ultimately, leading to one of the clinical manifestations discussed previously (Pulvertaft and Hoyle, 1960).

## 2 Current and emerging medications for the treatment of leishmaniasis

### 2.1 Chemotherapy

Pentavalent antimony was once thought to be the first-line pharmacological treatment for leishmaniasis; however, it is now known to cause cardiotoxicity, cirrhosis, pancreatic toxicity, and resistance risks (Frézard et al., 2009). Paromomycin was found to be helpful for Indian patients with VL, but less effective for Sudanese patients (Sundar and Olliaro, 2007). Amphotericin B (and lipid formulation) emerged as the second-line therapy as a result of this drug’s developing resistance (Pradhan et al., 2022). Since then, miltefosine has been used in VL and CL. Its advantages include being an oral medicine with good efficacy and a brief course, but its drawbacks include teratogenicity and drug resistance. Miltefosine is still a significant alternative in VL chemotherapy, even though its efficacy as a monotherapy has declined in recent years, especially when combined with other medications (Sundar et al., 2007). Repurposing of available drugs for leishmaniasis includes amphotericin B, miltefosine, paromomycin, and pentamidine (Andrade-neto et al., 1996). Leishmaniasis has also been investigated using azole antifungals, and itraconazole was found to be more effective than fluconazole and ketoconazole at preventing the growth of most *Leishmania* strains (Alrajhi et al., 2002).

### 2.2 Combination chemotherapy

Combination chemotherapy has been developed to prevent drug resistance, enhance compliance, decrease the length of treatment, and hence lower the cost of therapy. The other combinations are miltefosine plus paromomycin, miltefosine plus liposomal amphotericin B, and sodium stibogluconate/meglumine antimoniate plus paromomycin (Sundar & Olliaro, 2007), (Omollo et al., 2011).

### 2.3 Local therapies

Local therapies have been proven to be safe alternatives to systemic drug administration for CL-specific conditions. Thermotherapy, cryotherapy, and photodynamic therapy (PDT) have all been tested in CL. PDT uses topically administered aminolevulinic acid (ALA) or methyl-aminolevulinate, followed by laser or intense pulsed light irradiation. A few mechanistic investigations have focused on the ideas underlying the use of PDT for treatment of CL (Akilov et al., 2007). Eradication of host cells is the mechanism through which ALA-PDT exerts its anti-leishmanial effects for CL. Topical ALA-PDT is not suggested in clinical practice because of insufficient data (Tsai et al., 2002). At temperatures below 0, cryotherapy destroys infected cells, kills amastigotes, and modifies cell membranes to produce intracellular and extracellular ice crystals. Cryonecrosis results in the release of antigenic compounds that stimulate immune responses and repair further lesions. Since chemotherapy has several drawbacks, cryotherapy may be a useful alternative for treating CL. It has shown excellent response in patients with skin lesions ranging in size from 10 to 30 mm, those with fewer lesions, and those in whom the development was under 3 months. Cryotherapy and intralesional sodium stibogluconate were highly efficient, resulting in 100% healing of CL lesions. CO_2_ laser administration and thermotherapy based on the principle of directly destroying the *Leishmania* parasites is a simple way to deliver external heat on infected tissues, causing damage to specific areas with parasitism (Valencia et al., 2013). Heat sensitivity prevents *Leishmania* species from growing or surviving in environments hotter than 39°C. Thus, thermotherapy has been considered a possible treatment for CL lesions. Patients with CL have tried radiofrequency (RF) therapy, a type of thermotherapy. The cure rate for thermotherapy administered once every 3 weeks was 73%, whereas the cure rate for thermotherapy administered once every week was 81% (Sadeghian et al., 2007).

### 2.4 New developments in the treatment regimen of leishmaniasis

#### 2.4.1 Immunomodulators

Immunotherapy is a well-established treatment for leishmaniasis, and immunomodulators may be developed to achieve this goal under the hypothesis that a non-protective anti-leishmanial immune response could be transformed into a protective phenotype. The most promising method, when immunomodulators are considered, is therapeutic vaccination, which is based on cytokines that encourage macrophages to eradicate *Leishmania* parasites (Nicholls et al., 2010). IFN-γ immunotherapy for VL patients increased parasitological control and improved the therapeutic effectiveness of traditional pentavalent antimony (Sbv) therapy, enabling a more than 80% cure rate (Robledo et al., 1999).

#### 2.4.2 Nanotechnology as a drug delivery system

The field of drug discovery and design has undergone a revolution primarily because of nanotechnology, which has additionally proven to be a potential tool for parasitic diseases. It has managed to invent nanoparticles, as carriers, for drug delivery; liposomal formulation of amphotericin B reduces its toxicity profile (Date et al., 2007). Nanotechnology has significantly enhanced the conventional leishmanization procedure by inducing a Th1-type immune response in BALB/c during *L. major* infection using a liposome–protamine–DNA nanoparticle with immunostimulatory CpG (Alavizadeh et al., 2012), (Fakhraee et al., 2016). Nano-liposomal formulation of 1,2-dioleoyl-3-trimethylammonium-propane (DOTAP) and soluble leishmanial antigen (SLA) causes protection against *L. major* infection and enhances Th1-type immune response. Modified DOTAP, a second-generation vaccine candidate that has been reported as a conjugated nanoparticle with amastigote class I nuclease, makes for a promising candidate for the development of a CL vaccine (Fakhraee et al., 2016). By increasing its therapeutic efficacy, nanotechnology has thereby pioneered the door for the development of vaccines and medications (Sundar and Singh, 2018).

#### 2.4.3 Phytotherapy

In the past few years, phytotherapy has grown in popularity as researchers look for inexpensive anti-leishmanial treatments. The use of Kalanchoe pinnata, with components including triterpenes, sterols, and flavonoids, indicate substantial antileishmanial activity, but significant research in the field remains to be undertaken (Da Silva et al., 1995). Naphthoquinone is another class of secondary metabolite from plants with potential anti-leishmanial activity. Plumbagin, a naphthoquinone, has been reported to inhibit trypanothione reductase from *L. donovani* and induces mitochondria-mediated cell death (Awasthi et al., 2016).

#### 2.4.4 Target identification in signaling cascade of leishmaniasis

Recent research has shown that important proteins and macromolecules like lipids are great pharmaceutical targets by analyzing several metabolic as well as immunological signaling networks. While targeting metabolic pathways, identification of homology between the host and the parasite proteins is still the primary therapeutic target issue. Blocking energy metabolism remains a choice of target (Verlinde et al., 2001). Since Kreb’s cycle, glycolysis, and oxidative phosphorylation occur in the glycosome and mitochondria, these structures serve as the primary energy production houses. Interruption between any steps of glycolysis or Krebs’s cycle arrest the energy flux, leading to parasite killing. The potential drug target chalcone was discovered to be an anti-leishmanial prospective pharmacological target that targets the ultrastructure and functions of mitochondria, and later its ability to block fumarate reductase (Zhai et al., 1999), (Zhai et al., 1995).

Endochin-like quinolones (ELQs) have been demonstrated to be harmful to *L. donovani* and *L. mexicana* amastigotes, and hydroxynaphthoquinone buparvaquone is a more effective inhibitor of electron transport, ATP synthesis, and parasite multiplication, raising concerns about targeting cytochrome bc1 as a potential therapeutic strategy (Ortiz et al., 2016).

Polyamine metabolism is still an important prospective pathway for medication development. The first enzyme in the polyamine biosynthesis pathway is arginase (E. R. Da Silva et al., 2015), whereas ornithine decarboxylase (Boitz et al., 2009), S-adenosylmethionine decarboxylase (Brun et al., 1996), polyamine oxidase (Baumann et al., 1990), trypanothione synthetase, trypanothione reductase, tryparedoxin peroxidase, and deoxyhypusine synthase act as potential drug targets (Khan, 2007), (Wyllie et al., 2004).

**FIGURE 2:**
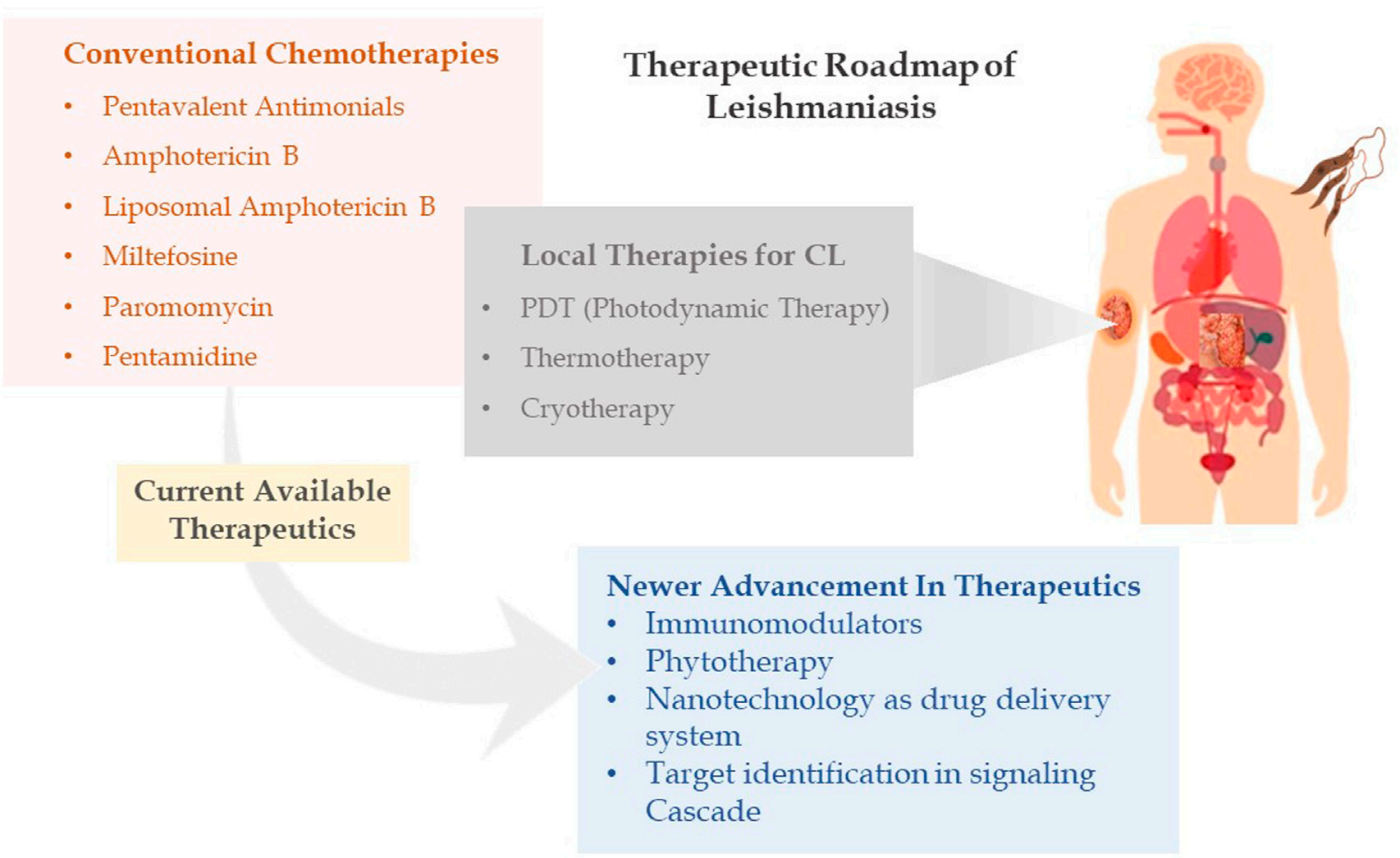
Therapeutic roadmap of leishmaniasis illustrating conventional chemotherapies, local therapies, and newer advancement in therapeutics.

It has long been understood that the generation of sterols is indispensable for cellular health and preservation of cell structure. Ergosterol and 24-methyl sterol are the most important sterols in trypanosomatids for growth and viability. As a result, the sterol and fatty acid metabolic pathways are appealing therapeutic targets. The mitochondria are the primary sites for fatty acid metabolism, with 3-hydroxy-3-methylglutaryl-CoA (HMG-CoA) reductase from *L. donovani* reported as a potential therapeutic target (Dinesh et al., 2014). Fatty acyl-CoA ligase affects cellular lipid homeostasis and has been found to be differently regulated in antimony-resistant *L. donovani*, confirming its potential as a therapeutic target (Kaur et al., 2011). Other than this, enzymes involved in sterol biosynthesis including farnesyl diphosphate synthase (Docampo & Moreno, 2008), sterol methyl transferase (Lorente et al., 2004), sterol 14 alpha-demethylase (McCall et al., 2015), and squalene synthase (Rodrigues et al., 2008) are reported as drug targets involved in chemical inhibition of the biosynthesis pathway. Immune signaling, energy metabolism, polyamine pathways, and fatty acid metabolism are examined by researchers to highlight potential molecules in the signaling cascade. Despite phenomenal research being conducted to create therapeutic targets for leishmaniasis, chemotherapy still serves as the cornerstone of effective treatment (Figure 2 depicting the treatment regimen of leishmaniasis). The details of therapeutics in leishmaniasis are listed in Table 1.

**TABLE 1:** Available therapeutics and newer development in leishmaniasis.

Therapeutics	Therapy	Treatment	Reference
Current available therapies	Chemotherapies	Pentavalent antimonials	Frézard et al. (2009)
		Amphotericin B	Kumar Saha et al. (1986)
		Liposomal amphotericin B	Pradhan et al. (2022)
		Miltefosine	Croft et al. (2006)
		Paromomycin	Sundar and Chakravarty (2008)
		Pentamidine	Lai A Fat et al. (2002)
	Local therapies	Photodynamic therapy	Akilov et al., 2007, Tsai et al., 2002
		Thermotherapy	Sadeghian et al. (2007)
		Cryotherapy	(Leibovici and Aram, 1986)
Roadmap in therapeutics development	Novel development in therapeutics purpose	Immunomodulators	Nicholls et al., 2010, Robledo et al., 1999
		Phytotherapy	Da Silva et al., 1995, Awasthi et al., 2016
		Nanotechnology	Date et al., 2007, Alavizadeh et al., 2012, Fakhraee et al., 2016
		Crucial proteins in the signaling cascade	Verlinde et al., 2001, Zhai et al., 1999, Ortiz et al., 2016

Researchers and international health organizations are compelled to develop novel strategies to combat and control this serious, untreated disease leishmaniasis due to the emergence of drug-resistant strains (Sundar, 2001), high toxicity, co-infections like HIV/*Leishmania* spp., limited chemotherapeutics available for disease treatment, and low investment for the discovery/development of new drugs for a particular disease. Thus, to investigate novel therapeutic options to effectively eradicate parasites, an understanding of host–parasite interactions is a prerequisite. Autophagy is found to be one of the crucial fundamental processes involved in leishmaniasis. Here, in this review, we elucidate the role of autophagy in leishmaniasis.

## 3 Autophagy

Autophagy is a climacteric cellular catabolic process to maintain cellular homeostasis, and it is conserved in a hierarchy from yeasts to mammals. The term autophagy was coined by Christian de Duve in 1963, but it remained a biological enigma until the early 1990s (De_Duve, 1974- Lysotropic. pdf). Furthermore, in the early 1990s, Yoshinori Ohsumi, an Assistant Professor at Tokyo University, studied autophagy using the budding yeast *Saccharomyces cerevisiae* as a model system and revealed some basic phenomena of the autophagy. He also discovered gene autophagy 1 (APG1), which is responsible for autophagy in yeast, and reported 15 more APG genes responsible for autophagy in the eukaryotic cell yeast. Autophagic genes were further identified in yeast and other species, and this specific group of proteins is abbreviated as ATG (Tsukada and Ohsumi, 1993), (Matsuura et al., 1997). All cells engage in low-level basal autophagy to carry out homeostatic functions like protein and organelle turnover. When cells produce intracellular nutrients and energy, such as during deprivation, growth factor depletion, or high bioenergetic demands, it is quickly activated. Furthermore, autophagy is activated when cells are ready to undergo structural remodeling, such as during developmental transitions, or when they need to get rid of potentially harmful cytoplasmic elements, such as under oxidative stress, infection, or the buildup of protein aggregates. Regulation of autophagy is significantly influenced by nutritional status, hormonal factors, and other factors like temperature, oxygen concentrations, and cell density (Klionsky, 2007), (Maiuri et al., 2007).

To date, collectively 41 types of proteins are known to play an important role in autophagy in different groups on the basis of their functions. These proteins are classified into six functional groups: ATG1-kinase/ULK1 complex, phosphatidylinositol (PI) 3-kinase complex (PI3K), membrane protein ATG9, ATG2–ATG18 complex, ATG16L conjugation system, and ATG8 conjugation system (Mizushima et al., 2011). These proteins and their homologs are well-studied in yeast as well as in mammals (C. He & Klionsky, 2009).

## 4 Mechanism of autophagy in mammals

Initiation of autophagosome formation in mammals is a complicated process, orchestrated by three major proteins ULK1 (unc-51-like kinase 1) ATG13, FIP200 (focal adhesion kinase family interacting protein of 200 kDa), and ATG101 combined to form the ULK1 complex. Succeeding autophagy induction, the ULK1 complex translocates to the autophagophore membrane or autophagy initiation site and further regulates PI3K complex recruitment over the autophagophore. This PI3K complex includes the VPS (vacuolar protein sorting) 34 (VPS34) complex, class III phosphatidylinositol 3-kinase VPS34, Beclin-1, VPS15, and ATG14L (ATG14-like), which govern the production of the phospholipid phosphatidylinositol 3-phosphate (PI3P) at the autophagophore. WIPI (WD repeat domain phosphoinositide interacting protein 2) and DFCP1 (double FYVE-containing protein 1) are recruited at the PI3P binding site and contribute to autophagophore expansion (Zachari & Ganley, 2017), (Bento et al., 2016). PI3P acts as a crucial component where numerous proteins are recruited and helps in autophagosome formation. ATG2 interacts with ATG18, which acts as a PI3P-binding partner, and forms a complex that further translocates to autophagophores and contributes to autophagosome biogenesis (Kotani et al., 2018).

**FIGURE 3:**
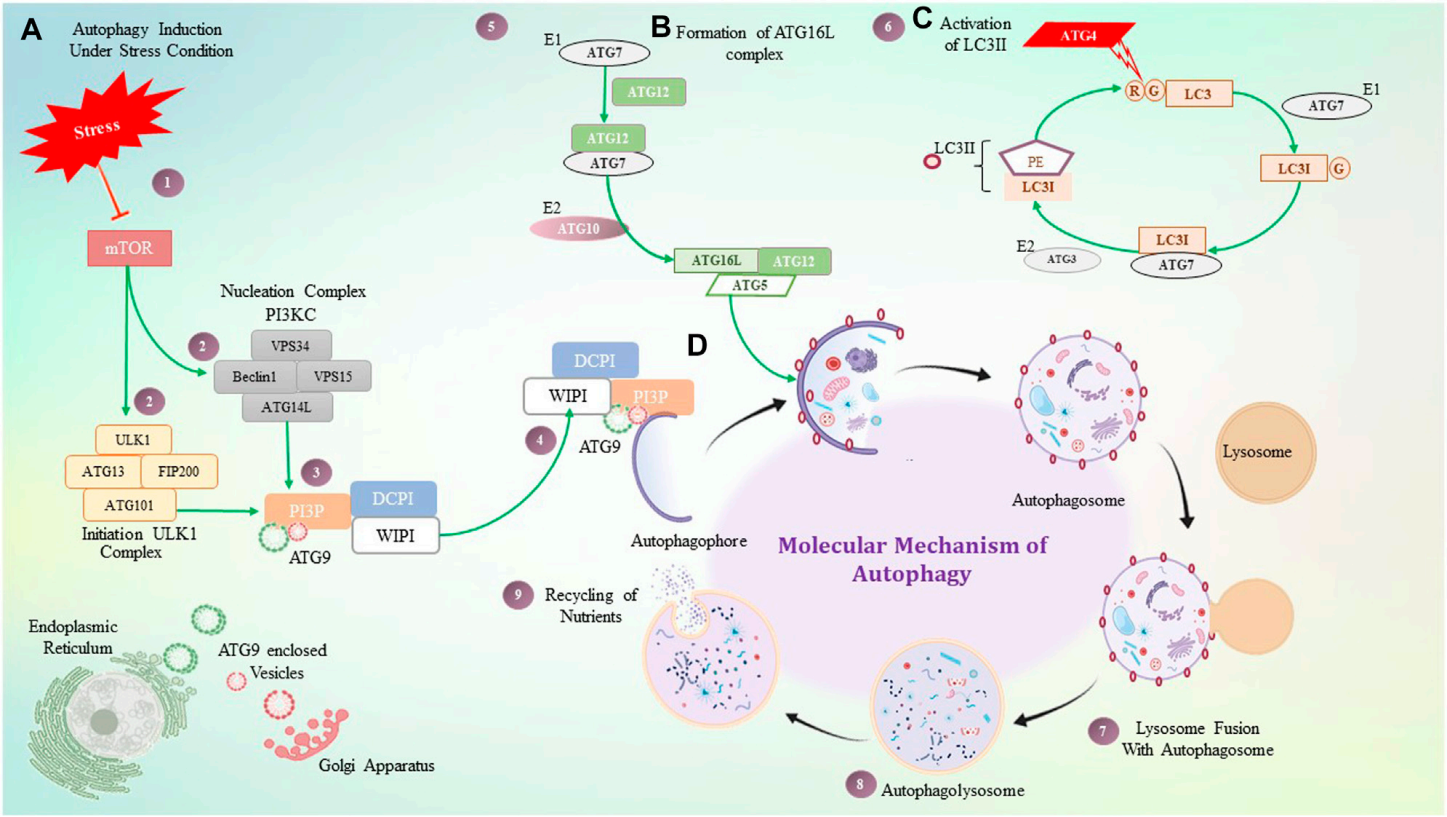
Molecular mechanism of autophagy. **(A)** Under stressful conditions, mTOR induces autophagy by activating the ULK1 and PI3KC3 complex, which then activates PI3P. PI3P is interacting with ATG9, DCPI, and WIPI proteins, which help with autophagosome elongation. **(B)** ATG16 forms a complex non-covalently with the ATG12 and ATG5 conjugate, where ATG7 plays a role as an E1-like enzyme. **(C)** ATG4 is a cysteine protease that cleaves LC3 protein at the glycine residue, and with the help of ATG7- and ATG3-like enzymes, it binds to PE which is termed LC3II. LC3II gets stabilized on the autophagosome with the help of the ATG16L1 complex. **(D)** Step-by-step diagrammatic description of autophagy shows the initial formation of the autophagosome membrane, followed by engulfment of damaged cellular components, their fusion with the lysosome, and the formation of the autophagolysosome.

In participation with the PI3K complex, ATG9 is playing a crucial role in lipid accumulation on the autophagophore and its expansion; ATG9 is a multi-spanning membrane protein (Geng et al., 2008), (Webber & Tooze, 2010), (Kotani et al., 2018). The exact molecular role of ATG2, as well as ATG9, is still mysterious, but directly or indirectly, they contribute to the PI3K complex for autophagosome initiation and expansion. Along with these complexes, two major autophagy-specific ubiquitin-like protein conjugation systems play a crucial role in autophagosome formation and maturation. It begins with the ATG16L conjugation system that consists of the ATG12–ATG5 conjugate covalently linked by ubiquitin-like enzymes ATG7 and ATG10 that act as E1 and E2-like enzymes, respectively. In addition, for localization of this conjugate on the autophagosome membrane, ATG16L is required. The ATG12–ATG5 conjugate and ATG16L that interact non-covalently are recruited on the membrane, whereas ATG5 mediates the interaction leading to autophagosome expansion (Romanov et al., 2012), (Noda et al., 2013). The ATG12–ATG5 conjugate has been shown to mediate LC3II–PE complex formation, while the ATG16L complex is observed to stabilize this complex on the membrane. In addition to acting as a marker for autophagy, the LC3II–PE conjugate is an essential step in the production of autophagosomes. Formation of the LC3II–PE conjugate is mediated by ATG4, which acts as a cysteine protease and cleaves ATG8 from the C-terminus at arginine and exposes the glycine residue and allows it to interact with PE (phosphatidyl ethanolamine) (Maruyama and Noda, 2018). This glycine-exposed LC3 and PE interaction is facilitated by ATG7, an E1-like enzyme that transfers LC3I to an E3-like enzyme and subsequently transfers LC3I to the membrane lipid PE, and LC3II complex translocates on the autophagosome. Autophagosome maturation takes place by various ATG protein complexes, as shown in Figure 3.

## 5 Role of autophagy in pathogenesis

Autophagy processes are involved in strategies to eliminate invasive pathogens through the production of reactive oxygen species, adjustment of vital nutrients and cations, destruction by proteolytic enzymes (Galán and Cossart, 2005), and by playing a crucial role in host–pathogen interaction, especially in intracellular pathogenesis. Research conducted over the last few years suggests that autophagy is one of the most astonishing intracellular host cell defense machinery tools that pathogens must spread during cell invasion. During infection, autophagosomes engulf intracellular pathogens; this process is known as xenophagy since it results in the eradication of foreign material. Pathogens are engulfed during autophagy and bind to the autophagosome membrane protein LC3 (light-chain 3)/Atg8 (autography-related protein 8).

To establish a persistent infection, most intracellular pathogens manipulate the autophagy pathway at the molecular level (Campoy and Colombo, 2009). Toll-like receptors (TLRs) that identify pathogen-associated molecular patterns induce autophagy during infection and allow autophagy to kill the pathogen. TLR signaling cascade and autophagy are two innate defense mechanisms that allow intracellular parasite clearance (Delgado et al., 2008). TLRs recognize conserved microbial components and induce a variety of antimicrobial activities, such as xenophagy via Myd88 and TRIF interacting with Beclin 1 (Campos-Salinas et al., 2013), (Shi and Kehrl, 2008). Autophagy also gets induced by cell-to-cell signaling. In human cells, IFN-γ stimulates autophagy through IRGM1, whereas CD40 ligation causes autophagy through PI3K and Rab7, enabling cells to fight intracellular infections like *Toxoplasma gondii* (Andrade et al., 2006).

Several molecules, including Nod-1 and Nod-2, recognize microbial antigen peptidoglycans and target bacteria for xenophagy (Cooney et al., 2010), (Travassos et al., 2010). However, many pathogens escape the phagosome after phagocytosis and proliferate in the cytosol. Microbes can also promote xenophagy via a variety of cell stress mechanisms, whereas *T. gondii* increases intracellular calcium levels to promote autophagy (Singh et al., 2009). Studies of Group A streptococci (GAS) also show evidence of autophagy. Getting rid of GAS (escapes from endosomes by encoding the hemolytic toxin streptolysin O) is a great illustration of autophagy, where ATG5 associates with ATG8/LC3 to form autophagic vacuoles for clearance of GAS (Nakagawa et al., 2004).

In the extensively studied pathogen *Mycobacterium tuberculosis* (Mtb), the removal of bacteria by autophagy, IFN (interferon)-induced IRGs (immunity-related p47 GTPases), and the overexpression of LRG-47 (one of the 23 IRGs in mice) promote autophagosome formation and autophagy-dependent clearance of phagosomes containing Mtb in mice (Gutierrez et al., 2004). Both mouse IRGs and autophagy can target the phagosomes that *T. gondii* uses for replication during toxoplasmosis. In contrast to the macroautophagy upregulating effect found for IRGs during Mtb infection, the mouse IRGs, IIGP1 and IGTP, appear to disrupt the *T. gondii*-contained PV (parasitophorous vacuole) membrane and even the parasite’s membrane, exposing it for destruction via macroautophagy (Tang et al., 2006), (Romanov et al., 2012). An effective illustration of evading autophagosome engulfment is *T. cruzi* (the causative agent of Chagas disease) infection to mammalian cells and activation of a signal transduction cascade that results in the establishment of a parasitophorous vacuole. Lysosomes have a significant impact on how *T. cruzi* infections progress, according to studies. The *T. cruzi* vacuole is decked with the host cell autophagic protein LC3. Fasting or pharmacologically induced autophagy increased the number of infected cells dramatically before infection, while inhibitors of this process significantly decreased the invasion, showing that mammalian autophagy is an important process that favors colonization of *T. cruzi* in the host cell (Alvarez et al., 2008). Skin lesions from BALB/c mice having CL also exhibit autophagy. In cultured macrophages, autophagy inhibitor 3-methyladenine (3 MA) decreased the infection index, while autophagy-inducing agents like rapamycin or fasting had no effects. This finding suggests that one function of autophagy is to provide nutritional support during infection (Cyrino et al., 2012).

In a comparative study of *L. major* and *L. amazonensis*, both species of infected macrophages demonstrated an increase in the LC3-II/Act (autophagic marker) ratio after 24 h, although treatment with autophagic inhibitors had no discernible effect on the parasite burden or infection rate. It is noteworthy to observe that pharmacological autophagy inducers increase parasite survival, whereas pharmacological autophagy inhibitors have no effect on it (Dias et al., 2018).

Researchers have become more interested in autophagy in leishmaniasis despite contradictory findings concerning the importance of autophagy in leishmaniasis. The reason is autophagy has been evidenced to be a therapeutic target in several intracellular pathogeneses, inflammatory illnesses, metabolic disorders, diabetes, cancer, osteoporosis, and other conditions.

Literature on leishmaniasis focuses on the interaction between lipid metabolism and autophagy. Both autophagy and lipid metabolism are necessary for creation of new perspectives on the investigation of leishmanial therapy. We are aware of very few articles that discuss the significance of lipid metabolism and autophagy. In this review, we try to highlight these crucial processes concerning therapeutics.

## 6 Role of lipid metabolism in host–parasite interaction

### 6.1 Lipid droplets

According to earlier studies, *Leishmania* infection alters the cell’s lipid metabolic pathways, where lipid droplets play a critical role (Bouazizi-Ben Messaoud et al., 2017). Lipid droplets (lipid bodies, LDs) are dynamic organelles that have beneficial roles in modulating lipid metabolism, energy homeostasis, signaling, membrane trafficking, and inflammation. Lipid droplets are roughly spherical structures consisting mostly of triacylglycerols and sterol esters which lack a traditional bilayer membrane but are surrounded by a monolayer of phospholipids and cholesterol as well as a few related proteins (Vallochi et al., 2018).

LDs are organelles that are closely associated to the ER. According to the first and most widely used theory of how LDs formed, these organelles originate from the accumulation of recently synthesized lipids within the double layer of the ER membrane, which then bud off into the cytoplasm once they reach a crucial size (S. Martin and Parton, 2006). Due to their varied protein and lipid components and involvement in essential cellular activities, LDs have become an important specialized and inducible cytoplasmic organelle. Beyond managing lipid metabolism, LDs additionally play a role in cell signaling, immunological activation, membrane trafficking, and formation and secretion of inflammatory mediators.

**FIGURE 4:**
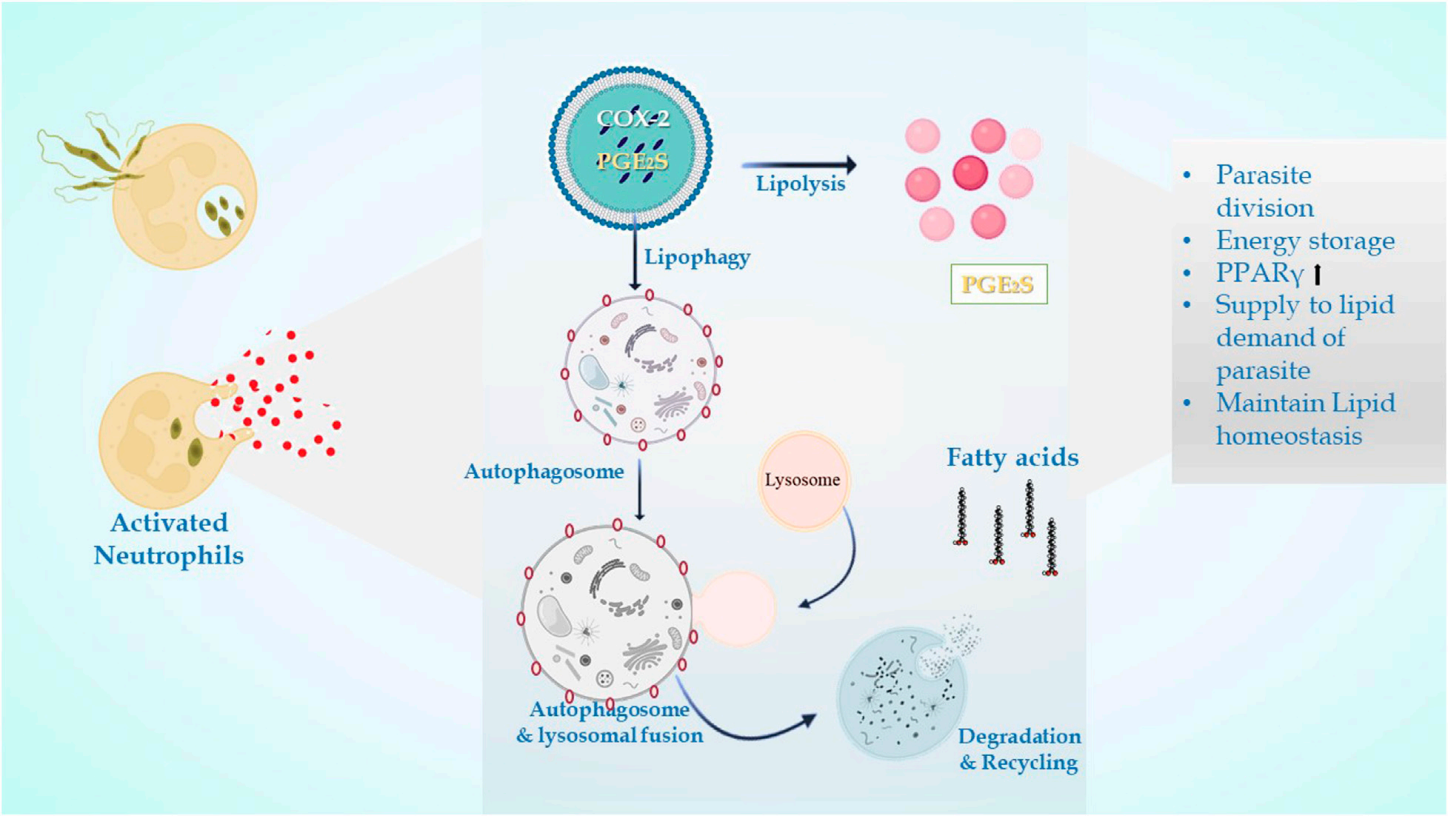
In leishmanial infection, lipid droplets are induced in the host during infection in response to provide lipids for parasite survival and for their differentiation. Within the LDs of stimulated cells acquired under infectious circumstances, the key eicosanoid-forming enzymes, cyclooxygenase, 5-lipoxygenase (5-LO), 15-LO, and PGE2 synthase were discovered. The synthesis of prostaglandin E2 aids in parasite survival. The production of signals that cause LD formation, fatty acid intake, lipid storage, and inflammatory response has been identified to involve PPARγ.

The LDs in infectious disorders also point to roles that go beyond simple interactions with numerous viral proteins or bacteria; they can take part in significant immune system cellular processes. Recently, researchers have been concentrating on the downstream pathways connected to the production of LD once an infection occurs (Figure 4). It has been determined how signaling pathways regulate the expression of genes linked to lipid influx/efflux and *de novo* synthesis during infectious processes. In accordance with a recent report, bacterial components may alter the expression and functioning of PPARγ. The lipid-activated nuclear receptor family includes PPARγ, a member of which directly controls a few genes involved in fatty acid intake, lipid storage, and inflammatory response. PPARγ has emerged as a crucial regulator of lipid metabolism and inflammatory genes in macrophages and dendritic cells (Odegaard et al., 2007). LD generation during protozoan parasite infection is mainly mediated by TLR2. TLR2-dependent LD formation was triggered in macrophages by *T. cruzi* infections. Macrophages’ absorption of apoptotic cells enhanced LD formation and PGE2 generation through TGFβ-signaling (D’Avila et al., 2011).

### 6.2 Significance of lipids and lipid droplets in *Leishmania* parasite

There is a huge involvement of lipids for the rapid multiplication of parasites and establishment of infection. *Leishmania* infection is efficient in modulating cholesterol metabolism in the host tissue in both acute and chronic patients (Johndrow et al., 2014). Cholesterol or ergosterols are essential components of plasma membranes found in lipid rafts and membrane microdomains due to their physiochemical properties. The available chemotherapeutic drug for leishmaniasis, amphotericin B, preferentially binds to ergosterol, causing the membrane’s osmotic integrity in target cells to be compromised (Chattopadhyay and Jafurulla, 2011), (Xu et al., 2014). Recently, quantified sterols of *L. major* through gas chromatography–mass spectrometry allowed (Yao and Wilson, 2016) comparing both qualitative and quantitative characteristics of sterols in infectious and non-infectious parasite forms.

The *Leishmania* parasite lacks a *de novo* mechanism for cholesterol production and thus must scavenge this lipid from the host environment (Biagiotti et al., 2017). According to reports, sphingomyelin degradation in the host acts as an enticing driver of *Leishmania* parasite infection (Martínez and Ruiz, 2019). Sphingolipids (SLs) are a group of cardinal membrane compounds that are produced abundantly in mammalian cells, in the form of sphingomyelin and glycosylsphingolipids, which do not exist in parasite cells (Ghosh et al., 2012).

Promastigotes synthesize most of their lipids, including glycerophospholipids, sterols, and sphingolipids, by *de novo* synthesis. Amastigotes, on the other hand, are able to carry out *de novo* synthesis but obtain most of their lipids from the host. The change in *Leishmania* from rapidly replicating promastigotes to slowly growing, metabolically dormant amastigotes is reflected in the transition from *de novo* synthesis to salvage (Zhang, 2021). Phosphatidylcholine (PC), phosphatidylethanolamine (PE), and phosphatidylinositol (PI) are the three phospholipids most abundant in *Leishmania* promastigotes, with other phospholipids found in smaller amounts. In comparison to promastigotes, amastigotes have higher levels of phosphatidylserine and sphingomyelin and lower levels of PE and PI (Martínez and Ruiz, 2019). Additionally, the parasite’s non-infectious form is distinguished by having more unsaturated fatty acids than saturated ones and a significant generation of n-6 polyunsaturated fatty acids in comparison to n-3 forms (which is lowered in amastigotes) (Biagiotti et al., 2017).

It has been reported that the percentage of cholesterol almost doubled in the amastigotes, whereas the concentration of ergosterol decreases significantly (80%) (Bouazizi-Ben Messaoud et al., 2017). Cholesterol is scavenged by intracellular parasites from the host. As a source of energy storage, to maintain metabolic balance, and to escape the host reaction, intracellular parasites have their LDs. As *de novo* synthesis is absent in parasites, lipids needed by many intracellular parasites are obtained from host LDs. Intracellular parasites have acquired methods to entice host LDs to their phagosome compartment and engulf them whole (Melo and Dvorak, 2012). It is unclear how lipids are incorporated by *Leishmania* parasites at the cellular and molecular levels.

### 6.3 Interplay between lipid metabolism and autophagy

**FIGURE 5:**
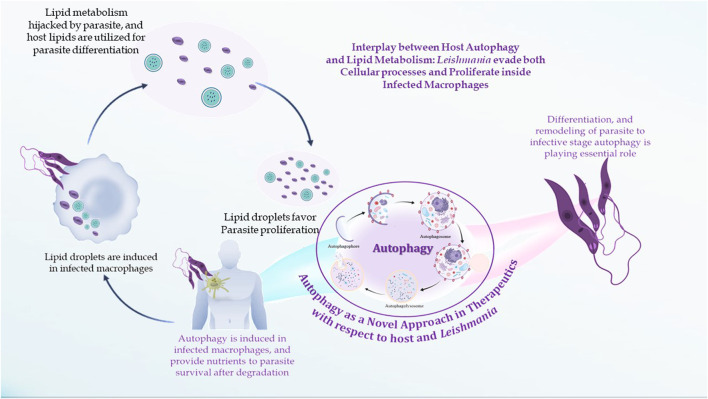
Interplay between lipid metabolism and autophagic processes.

Murine macrophages infected with *Leishmania* exhibit increased cholesterol absorption and triacylglycerol production, resulting in the development of lipid droplets near or inside parasitophorous vacuoles (PVs) (Rodríguez et al., 2017). Amastigote forms replicate inside the host macrophages within PVs, specialized membrane-bound organelles of the endocytic pathway with late endosomal/lysosomal characteristics. PV morphology differs depending on the species of *Leishmania*. Large communal PVs characterize infection with both *L. mexicana* and *L. amazonensis* and are formed by the fusion of small individual vacuoles with compartments of the endocytic pathway. According to several studies, host cell macromolecules are transported to the PV lumen and endocytosed by the parasite. PVs get cytosolic macromolecules from *L. mexicana*-infected macrophages by a process resembling host cell autophagy (Pinheiro et al., 2009). The interrelationship between autophagy and lipid metabolism is termed macrolipophagy (Singh et al., 2009), as autophagy regulates lipid content. Singh et al. observed that inhibition of autophagy increases TGs and LDs *in vitro* and *in vivo,* whereas loss of autophagy decreases TG breakdown and LC3 associated with LDs. There is also a reverse relationship that suggests an abnormal increase in intracellular lipids impairs autophagic clearance, based on decreased LD/LAMP1 co-localization, absence of autophagic upregulation in hepatocytes cultured with lipids, and diminished association of autophagic vacuoles with LDs in response to starvation in HFD-fed mice. This interrelationship shows that decreased autophagy promotes lipid accumulation, which then further suppresses autophagic function, thereby additionally increasing lipid retention (Figure 5).

## 7 Autophagy in leishmaniasis

### 7.1 Autophagy induction by *Leishmania* in the host

Studies have demonstrated that depending on the type of parasite and the host interaction, autophagy can either support infection or facilitate parasite expulsion. To date, studies have examined the involvement of autophagy in *Leishmania* infection. Although it has been repeatedly demonstrated that the *Leishmania* parasite induces autophagy in different cells, but how autophagic modulation affects *Leishmania* survival is still conflicting.

Earlier studies have shown that *L. mexicana* has developed access to endosomes, phagosomes, and autophagosomes as sources of host cell material; such a strategy might offer the parasite two different adaptive benefits. In the beginning, it offers an additional supply of nutrients. Furthermore, PVs are part of the host’s route for processing and presenting antigens (Schaible et al., 1999). For the first time, Mitroulis, Ioannis, et al. discovered induction of the autophagic machinery (i.e., LC3B conversion) during acute natural bone marrow infection by *L. donovani* under real-time circumstances, although the role of autophagy in parasites was discovered earlier. For obtaining essential nutrients, *Leishmania* species trigger autophagy. The expression of the autophagy genes Atg7 and LC3, as well as the LAP-like accumulation of LC3, around the parasite vacuoles, appears to be related to the severity of the *L. infantum* infection (Esch et al., 2015). Nutrient deprivation is a potential inducer of autophagy, *L. amazonensis* was administered to BALB/c macrophages, and durations of 1–24 h of amino acid and serum fasting were used to induce autophagy. The result demonstrated the elevation of positively stained vacuoles for MDC (monodansylcadaverine), a marker of autophagy. The intracellular burden of *L. amazonensis* in BALB/c macrophages increased in response to inducers rapamycin and glucagon. In response to starvation, the intracellular load of *L. amazonensis* increases up to 2–12 h without altering macrophage viability, but it unexpectedly decreases at 24 h by loss of macrophage viability. Altogether, our findings suggested that, following the onset of autophagy, intrinsic host cell factors regulate the course of infection (Pinheiro et al., 2009). Also, it was highlighted that one function of autophagy may provide the parasite with nutritional support, as 3-methyladenine (3 MA), the autophagy inhibitor, decreased the infection index, while autophagy inducers rapamycin or fasting had no effect (Cyrino et al., 2012).

Autophagy stimulation was observed by IFN-γ or fasting-boosted *L. amazonensis* infection in BALB/c mice, but not by *L. major* infection. As *L. amazonensis* amastigotes do not co-localize with IFN- γ-induced, double-membrane vacuoles, starvation or autophagy-inducing agents may make it favorable for the parasites to be consumed and proliferate (Pinheiro et al., 2009). Not only in macrophages, *Leishmania* can also induce autophagy in neutrophils. This study uncovered that canonical autophagy was prevalent in *Leishmania* infection after 3 h and that *L. donovani* infection encouraged a time-dependent enhanced autophagy, responsive to blocking by 3-methyladenine but sensitive to ULK1/2 inhibition only after 3 h (Pitale et al., 2019). Thomas et al. (2018) reported that in THP-1 cells, knockdown of Atg5 and Atg9 reduced intracellular *L. donovani* survival. On the other hand, *L. major* parasite load was increased by Atg5 knockdown in BALB/c and C57BL/6 macrophages (Franco et al., 2017). *Leishmania* fine-tunes the time and inhibits mTOR-regulated autophagy while concurrently enhancing parasite survival (Thomas et al., 2018).

Pattern-recognition receptor (PRR), TLR, is also responsible for induction of autophagy in infected macrophages as macrophages deficient in TLR3, 7, and 9; UNC93B1; or MyD88 failed to undergo *L. major*-induced autophagy (Franco et al., 2017). Matte et al., 2016 focused on LC3-associated phagocytosis, the non-canonical aspect of autophagy in leishmaniasis. During *L. major* infection, LC3 retention in the autophagosome is diminished, which occurs as a result of VAMP8, and NOX2 NADPH oxidase—required for the formation of ROS—is inhibited in a way that is dependent on the parasite surface metalloprotease GP63. Overall, the results showed that VAMP8 is important for LC3-associated phagocytosis, while GP63 is necessary for *L. major* survival. Beatriz R. S. Dias, in 2018, reported an increase in the LC3-II/Act ratio upon infection with *L. major* and *L. amazonensis*.

Although LC3 requirement on parasitophorous vacuoles was enhanced after 24 h, no impact on parasite load or infection rate was imposed by autophagy inducers or inhibitors in infected macrophages. However, by reducing NO production, the inducer helps *L*. *major* to become more viable than *L. amazonensis*, whereas the inhibitor had no effect (Dias et al., 2018). In glomerulonephritis caused by *L. infantum*, elevated levels of the glomerular nucleotide-binding domain leucine-rich repeat-containing-like receptor family, pyrin domain-containing 3, and autophagosome-associated LC3 within glomeruli and tubules were observed to be indicative of the induction or response to glomerular deposition of immune complexes and antigen, according to immune-histopathology and transcriptional analysis (Esch et al., 2015). Apoptotic-like *Leishmania* uses the autophagic machinery of the host cells to inhibit CD4^+^ T-cell proliferation, which limits the parasite’s ability to survive intracellularly (Crauwels et al., 2015).

Several studies have examined how autophagic activation impacts the pathophysiology of *Leishmania* infection, but the interactions between the parasite and the host have produced contradictory and varying results. It is evident that more mechanistic research and explanation are needed for the precise model of interaction between *Leishmania* species and the host autophagy mechanism. Mechanistic insights into autophagy in disease, which differentiate the autophagic machinery in the host and in the parasite, are proven to be critical for a clearer understanding.

### 7.2 Role of autophagy in *Leishmania* parasite

Research on trypanosomatids has indicated the significance of autophagy in the differentiation and remodeling of the parasite under starvation, and it has been proposed that preventing autophagy might be a newer approach to battling parasite illness (Vanrell et al., 2017), (Besteiro et al., 2006), (Besteiro et al., 2007). As autophagy is an essential lysosomal network in response to nutrient deprivation, similar illustrations have been observed in parasite *L. major*. In *Leishmania*, autophagy is critical for maintaining cellular homeostasis, recycling damaged organelles, turnover of glycosomes via the Atg5 protein (Cull et al., 2014), and protein turnover (Besteiro et al., 2007). Moreover, metacyclogenesis, the process by which epimastigotes differentiate into metacyclic trypomastigotes, and polyamine metabolism both depend on autophagy (Vanrell et al., 2017).

The function of MVBs (multivesicular bodies) in *L. major* is examined by characterizing the leishmanial Vps4 homolog. A similar function of VPS4 was observed in *L. major* when the dominant negative mutant VPS4 (VPS4E235Q) accumulated the mutated protein around vesicular structures of the endocytic system and showed a defect in transport to the MVT lysosome, signifying conservation of the role of this protein in MVB architecture from the early branching kinetoplastid flagellate lineage to mammals. VPS4E235Q overexpressing *L. major* was impaired in differentiation, and their resistance to starvation proved the VPS4 and MVB compartment role in this process (Besteiro et al., 2006). The role of ATG4.2 in metacyclogenesis (Besteiro et al., 2006) as well as in virulence of the parasite (Williams et al., 2013) was reported. Other than ATG4, around 25 ATG8-like family proteins were discovered and designated as ATG8, ATG8A, ATG8B, and ATG8C. ATG8 has been revealed to form putative autophagosomes during differentiation and starvation of *L. major*; ATG8A has a role in starvation-induced autophagy, whereas ATG8B and ATG8C both have a distinctive subcellular location near the flagellar pocket, but the incidence of the GFP-labeled puncta recommends that they do not have a role in autophagy. In addition to this role, cysteine peptidase also plays roles in *L. major* similar to those of yeast and mammals, where ATG4.1 cleaves sessile glycine in ATG8, ATG8B, and ATG8C, while ATG4.2 is able to cleave ATG8A (Williams et al., 2009), (Williams et al., 2013). The parasite homolog of ATG12 has been found on parts of ATG8 containing puncta, indicating toward the ATG5–ATG12 conjugate in the parasite (Williams et al., 2009). The importance of ATG8 in parasite survival and infectivity was reported by Williams et al. (2012), and Giri and Shaha (2019) also reported the role of ATG5 in ATG8-dependent autophagy and mitochondrial homeostasis in *L. major*, which ultimately showed their significance in parasite virulence.

*Leishmania* parasite comprises various homologs of ATG proteins, even though the exact autophagic mechanism is not yet deciphered. Research points to a crucial function for autophagy and the protein it produces in the differentiation and infectivity of parasites. Thus, anti-leishmanial research may direct toward autophagy proteins in the parasite. Moreover, strategies developed by parasites to circumvent the host defense mechanism such as autophagy bring autophagy into the spotlight and give weightage to the enticing role of autophagy as an emerging target for therapeutics in leishmaniasis.

## 8 Autophagic process of the host as a therapeutic target in leishmaniasis

The effect of autophagy on parasite survival and its modulation made by the parasite for its own survival establishing the host autophagy pathway serves as a possible therapeutic target in the treatment of leishmaniasis. Available information highlighted a few of the protein molecules which can serve as a target for future therapeutic purposes against leishmaniasis.

*Leishmania*-infected human monocyte-derived macrophages (hMDMs) lead to stimulation of autophagy LC3-associated phagocytosis (LAP), which has an even sharper effect on decreasing proliferation by inhibiting CD4^+^ T-cell growth and proliferation of CD4^+^ T-cells, leading to a reduced intracellular parasite survival. This further implies that host cell autophagy modulates the T-cell response, enhances parasite persistence, and yet has no effect on the viability of the parasite. Apparently, *Leishmania* utilizes the autophagy mechanism of the host cells to inhibit T-cell proliferation and highlights the autophagy mechanism (Crauwels et al., 2015). CD4^+^ Th1 cell-mediated immunity is required for protective immunity against leishmaniasis (Okwor et al., 2014), (Peters et al., 2014) because the progression of the parasite is due to the exhaustion of T cells; however, the mechanism involved is not clearly understood (Esch et al., 2013).

Blocking PDL-1 signaling *in vivo* leads to reinstatement of protective type 1 response by both CD4^+^ and CD8^+^ T cells, and as a result, the parasite burden was significantly reduced. Inhibition of PDL-1 enhanced the production of TNF, IL-12, reactive oxygen species (ROS), and nitric oxide (NO) and significantly reduced parasite survival (Roy et al., 2019). Mechanistically, PDL-1 inhibition blocked the autophagy process (Robainas et al., 2017) hijacked by *Leishmania* to acquire host cell nutrients for their survival (Crauwels et al., 2015). The restoration of effector arms of protective immunity against leishmaniasis and subsequent parasite clearance can be achieved with anti-PDL-1 antibody therapy (Robainas et al., 2017).

The anti-pathogen properties of mammalian target of rapamycin (mTOR) have been proven, as well as its ability to prevent autophagy (Rangwala et al., 2014). *Leishmania* promotes the PI3K pathway in the host, and PI3K activation inhibits autophagy via enhancing mTOR (Ruhland et al., 2007). The ability of different mTOR inhibitors such as rapamycin, GSK-2126458, and KU-0063794 was evaluated by administering to infected BALB/c mice. In contrast to KU-0063794, rapamycin or GSK-2126458 significantly reduced the parasite load (Khadir et al., 2018) in the draining lymph node as well as the swelling of the footpad. Other studies demonstrated that pre-treatment of host cells with rapamycin to induce autophagy prior to infection was inhibitory to survival of parasites in a concentration-dependent manner, and once infection is established, the stimulation of autophagy by *Leishmania* confers the advantage of parasite survival.

Thomas et al. (2018) suggested that *Leishmania* infection delayed induction of host cell autophagy that is essential for the best possible intracellular survival. *L. donovani* boosted AKT activation to control the GSK-3/-catenin/FOXO-1 axis, preventing host cell apoptosis and immune response necessary for the survival of the parasite in macrophages (Gupta et al., 2016). Taken together, mTOR inhibitors or the PI3K/Akt pathway are used in therapeutic settings for managing leishmaniasis (Madeira Da Silva and Beverley, 2010), (Khadir et al., 2018), (Thomas et al., 2018).

*Leishmania* infection significantly induces the expression of SIRT1, which inactivated FOXO-1 through deacetylation, constitutively active FOXO-1 led to increased cell death, thereby suggesting that nuclear FOXO-1 might be inactivated (Gutierrez et al., 2004). SIRT1 knockdown led to increased apoptosis and significantly decreased parasite survival along with increased production of TNF-α, ROS, and NO. SIRT1 inhibitor sirtinol in infected mice shows a synergistic effect found with the PD-1 inhibitor. Although they are interconnected, the SIRT1/FOXO-1 axis is used by *Leishmania* to differentially regulate PD-1 signaling, and both pathways alone support intracellular parasite persistence (Roy et al., 2019).

The parasite uses the PERK/ATF4 pathway, which is crucial for maintaining protein homeostasis and establishing infection (Abhishek et al., 2018). Additionally, PERK signaling reduces translation and promotes ATF4 expression, which either promotes cell survival or causes cell death. Cytoprotective autophagy genes, ATG5 and ATG7, are known to be upregulated as a result of PERK/ATF4 pathway activation (Zheng et al., 2019), which favors cellular survival and parasite infection (Aoki et al., 2019). GSK2606414 previously used as an anti-cancerous drug was discovered through chemical inhibition tests to assess prospective drugs since it targets the kinase domain of PERK (Rangwala et al., 2014), and its therapeutic implications and role in leishmanial infection may substantiate it as a repurposed drug for treatment options for leishmaniasis.

PGE_2_ (prostaglandin E2) is responsible for the increment in parasite load in macrophages (Ribeiro-Gomes et al., 2004), and the physiological stimulation of autophagy promotes the intracellular survival of *L. amazonensis* by a process involving increases in the synthesis of lipid bodies and PGE2, as well as lower NO levels (Vallochi et al., 2018). Also, Martínez and Ruiz (2019) stated that lysosomes destroy LDs after autophagosomes sequester it during lipophagy. Growing evidence points to the involvement of LDs in the control of the autophagic process and their eradication by autophagy via lipophagy. Thus, blocking autophagy-mediated process via lipid droplets may serve as a newer area to combat leishmaniasis.

Recent research has shown that the host microRNA, MIR30A-3p, may regulate host autophagy following *L. donovani* infection. After *L*. *donovani* infection, MIR30A-3p expression is significantly increased in a time-dependent manner. MIR30A-3p mimic decreased and with antagomir increased the expression of BECN1 protein in *L. donovani*-infected macrophages, demonstrating the role of MIR30A-3p in autophagy in leishmaniasis (Singh et al., 2016). Autophagy and miRNAs are interconnected (Frank et al., 2015). BMDM showed intracellular amastigote clearance by autophagy in BMDM through mTOR phosphorylation-dependent counteracting mechanism after *in vitro* infection with *L. major*. Additionally, the study demonstrates a relationship between the infection-specific overexpression of (BCL2/adenovirus E1B 19 kDa protein-interacting protein) BNIP3 and (cathepsin-E) CTSE and autophagy-related protein 5 (ATG5). There is evidence for showing the involvement of miR-101c, miR-129, and miR-210 in RNA-level autophagy regulation during *L*. *major* infection and miRNAs have been linked to lipid metabolism, not in leishmaniasis but in other disorders (Latorre et al., 2017).

Altogether in *L. major*-infected host macrophages, autophagy is a tightly controlled cellular process at the RNA and protein levels (Frank et al., 2015), suggesting a role of RNAs in the modulation of autophagy in leishmaniasis.

A new era is emerging where people are trying to combat fatal situations with the use of microRNAs. So, in this review, we elaborate on the collaborative functioning of microRNAs with lipid-mediated autophagy, and the respective probability of incorporation of miRNA and lipids concerning autophagy in therapeutic approaches. The capacity of miRNAs to disrupt various signaling pathways and consequently alter the cellular response and the outcome of diseases is a current hotspot in medical research science (Yang and Wang, 2016), (Butterworth, 2018), (Barbu et al., 2020), (Gorabi et al., 2020), (Gholamrezaei et al., 2020). Gholamrezaei et al. (2020) have studied that the miR-155 inhibitor and mimic effect in *L. major*-infected macrophages can induce apoptosis and reduce parasite burden and hence highlighting it as an essential biomolecule that may be utilized as a therapeutic target against the disease. However, Taganov et al. (2006) have suggested that induction of miR-146a/b is triggered by those TLRs that mainly sense viral nucleic acids and localize in the intracellular area such as TLR2, TLR4, and TLR5 and proposed a role of a miR-146 in control of TLR and cytokine signaling through a negative feedback regulation loop involving downregulation of IRAK1 and TRAF6 protein levels. Pauley et al. (2010) have shown that the production of 5 cytokines/chemokines (IL-6, IP-10, IL-8, MCP-1, and IL-1b) was reduced after transfection of miR-146a mimic. Thus, it is demonstrated that miR-146a controls acute inflammation caused by LPS stimulation by limiting the production of cytokines after the initial innate response. O’Connell et al. (2007) Findings imply that the JNK pathway, which activates the AP-1 complex, is involved in miR-155 induction by both TLRs and TNF-α, presumably through transcriptional activation of the miR155 encoding BIC gene. Members of the let-7 family of miRNAs are well-represented in the data set in the study by Geraci et al. (2015). Most noticeable are the let-7a and let-7b dichotomous expression patterns, where only DCs and MPs infected with *L. donovani* display upregulation of those miRNAs, and cells infected with *L. major* were observed to downregulate the same miRNA (Geraci et al., 2015). For VL (Singh et al., 2016), results confirm that BECN1 is a potential target of MIR30A-3p in both THP-1 cells and HsMDMs infected with *L. donovani*. Di Loria et al. (2020) have studied that *Leishmania* organisms can disrupt the production of miR122, which will disrupt the metabolism of cholesterol and ensure its proliferation in the infected host, emphasizing its capacity to hamper lipid metabolism of the host.

The review clarifies that the invading pathogen’s main targets for modulating and maintaining infection in the host are the post-transcriptional and post-translational stages, miRNAs are significant contenders for considering their role in lipid-mediated autophagy.

While it is important to control autophagy brought on by infection to manage parasite survival, it is also important to pay attention to proteins that may serve as targets for autophagy in parasites. Numerous research studies attempt to uncover proteins or chemicals that might impair parasite diversity, infectiousness, or survival.

## 9 Exploration of the potential modulator to modulate autophagy in *Leishmania* parasite

Unsatisfactory leishmaniasis treatment encourages researchers to investigate more modern options, which now target the parasite’s functioning proteins or molecules. The heat shock protein 90 (Hsp90) is believed to be a commendable drug target against parasitic diseases (Debnath et al., 2014), (Stofberg et al., 2021), (O. Angel et al., 2013), (Silva et al., 2013), (De Andrade et al., 1992). 17-N-allylamino-17-demethoxygeldanamycin (17-AAG), a Hsp90 inhibitor, caused a leishmanicidal effect *in vitro* (Petersen et al., 2012) and *in vivo* (Santos et al., 2014) in leishmaniasis. In promastigotes, 17-AAG induces an autophagy pathway, leading to parasite death. Following treatment with 17-AAG, ∆atg5 parasites are less prone to cell death, which shows that autophagy is involved in inhibitor-induced parasite death, where ATG5 is playing an important role (Petersen et al., 2021).

Transporter proteins of the ABC subfamily, LABCG2, are essential for *Leishmania* virulence (Campos-Salinas et al., 2013). It is necessary for the externalization of phosphatidylserine, a macrophage invasion adaptation strategy. Additionally, it was shown that LABCG1 was a crucial component, cooperating with LABCG2 to control metacyclogenesis, infectivity, oxidative stress, and autophagy (Manzano et al., 2017). Studies on this transporter’s mutations showed that the parasite’s pathogenesis and virulence were both reduced. As a result, these transporters may be exploited as therapeutic targets, and in combination with them, it may be possible to overcome drug resistance (Kabra et al., 2020).

Natural indoloquinoline alkaloid cryptolepine (CLP) induces cellular dysfunction in *L. donovani* promastigotes, which leads to the contribution of initial autophagic response by showing monodansylcadaverine (MDC)-labeled autophagic vacuoles. However, cells later exhibit characteristics of cell death which may not be because of autophagy but due to prolonged intracellular stress due to CLP (Sengupta et al., 2011). The toxicity of quinoline derivative salts (QDS) was evaluated in mammalian cells, and the mechanism of action proved it as a promising compound (Calixto et al., 2018), while the *in vivo* effect of the qunoline derivative (AMQ-j) in BALB/c mice was evaluated (Antinarelli et al., 2018). QDS highlighted the anti-leishmanial impact of this substance by inhibiting the formation of autophagic vacuoles, which may have contributed to parasite death by impeding autophagic mechanisms in the clearance of damaged organelles. Additionally, an increase in ROS levels was observed, reinforcing that the induction of oxidative stress triggered the death of intracellular parasites (Calixto et al., 2018). On the other hand, the quinoline derivative (AMQ-j) exhibited several morphological and biochemical changes caused by AMQ-j, including intense mitochondrial swelling, collapse of the mitochondrial membrane potential, abnormal chromatin condensation, externalization of phosphatidylserine, accumulation of lipid bodies, disruption of the cell cycle, formation of autophagic vacuoles, and increase in acidic compartments (Antinarelli et al., 2018). These changes could be related to both QDS and AMQ-j and show a strong effect of autophagy-related and apoptosis-like processes against *L. amazonensis* and its anti-leishmanial activity (Calixto et al., 2018), (Antinarelli et al., 2018). Thiosemicarbazone molecule (BZTS) in synergy with the available drug miltefosine shows a significant increase in reactive oxygen and nitrogen species as a result of mitochondrial malfunction, leading to severe cell damage, an extensive autophagic process, and the ensuing apoptotic cell death in parasites by least damaging mammalian cells.

Thus, these findings support the synergetic impact on several metabolic pathways in leishmaniasis and encourage the notion of therapeutics toward metabolic pathways and further target identification (Scariot et al., 2017). Anti-leishmanial activity of apigenin *in vitro* and *in vivo* was described. Apigenin induced reactive oxygen species (ROS) generation, and the number of autophagosomes after the infection is suggestive of the involvement of host autophagy in macrophages infected with *L. amazonensis* (Fonseca-Silva et al., 2016). The role of ATG8 in *L. donovani* for its survival mechanism and infectivity showed it as a drug target. Molecular docking and molecular dynamic simulation studies show the enticing role of thiabendazole derivatives that impede the survival mechanisms by affecting ATG8 (Guhe et al., 2022), while virtual screening of natural ligand library had been performed against ATG8 of *L. donovani*, showing that urolithin-A which is formed by combinations of coumarin and isocoumarin stably blocked ATG8 (Rahman et al., 2022).

The attention of phenomenal research in leishmaniasis toward therapeutics considering limitations of current treatments is shifting toward autophagy. Targets identified in the *Leishmania* parasite and respective drugs are enlisted in Table 2. However, there is also a need for a novel approach due to the intricacy of the autophagy network, its connections to other metabolic pathways in the host, and homological conflicts between the presence of autophagy in the host and parasite. Given the complexity of the *Leishmania*–host relationship, further studies should concentrate on the genetic investigation of autophagic pathway alteration.

**TABLE 2:** Drugs showing anti-leishmanial effect by modulating the autophagy process and their respective targets.

S.N.	Targeted protein/pathway	Respective potential drug	References
1	Hsp90	17-AAG (17-N-allylamino-17-demethoxygeldanamycin)	(Petersen et al., 2012), (Santos et al., 2014)
2	LABCG2, transporter protein of the ABC subfamily	Transporter’s mutations or peptide designing against proteins	(Manzano et al., 2017), (Kabra et al., 2020)
3	Autophagic process which is crucial for dysfunction in *L. donovani* promastigotes	CLP (cryptolepine)	Sengupta et al. (2011)
4	Inhibition of formation of autophagic vacuoles	QDS (quinoline derivative salts)	Calixto et al. (2018)
5	Mitochondrial membrane potential and chromatin condensation	AMQ-j (quinoline derivative)	Antinarelli et al. (2018)
6	Mitochondrial functioning	BZTS (thiosemicarbazone molecule)	Scariot et al. (2017)
7	Host autophagy in macrophage	Apigenin	Fonseca-Silva et al. (2016)
8	ATG8 of *L. major* and *L. donovani*	Thiabendazole derivatives, urolithin-A	(Guhe et al., 2022), (Rahman et al., 2022)

## 10 Novel therapeutic strategies for leishmaniasis

The enormous growth in genomic data for pathogens, particularly the available genome sequence, has created a capacity to discover potential vaccines or chemotherapeutic targets. Strategies to accomplish clinical objectives have been made clear by the advent of functional genomics techniques, such as microarray and more recently deep sequencing technologies, as well as proteomics (Meissner et al., 2022). For instance, the proteomic technique is utilized for identifying the binding proteins for a given small molecule. Due to its arduousness and length, this strategy has not been very effective in identifying targets (Ivens et al., 2005). Additionally, in the post-genomic timeframe, these advancements enabled bioinformatics to arise, significantly accelerating the research process. A variety of confirmatory assays, computational algorithms, and high-throughput screening techniques all considerably aid in the identification and characterization of emerging, powerful drug targets (Tao et al., 2004), (Tang et al., 2006). In addition, several computational (*in silico*) technologies have also been designed.

In diseases caused by external pathogens like bacteria, viruses, and parasites, a comparative functional genomics from humans to the respective pathogen is found to be crucial for identification of novel targets (García-Lara et al., 2005). Genome availability is a key regulator when genetic techniques and bioinformatics approaches are taken into consideration for the understanding of the system. In the post-genomic era, genome information of various pathogens was deciphered, including *Leishmania* (Ivens et al., 2005), disclosing the haploid genome of *L. major* (Friedlin strain), where genes involved in host–pathogen interactions were highlighted. Based on the annotation released by the Wellcome Trust Sanger Institute and the genomic sequence of *L. major* (v5.2), the LeishCyc database was originally developed. LeishCyc is a platform for the analysis, interpretation, and visualization of *Leishmania* omics data (transcriptomics, proteomics, and metabolomics) in the context of metabolic pathways and offers a systematic approach to structuring the increasing information regarding *Leishmania* biochemical networks (Doyle et al., 2009). After the post-genomic era, various studies switched to computational approaches for discovering new drug targets as genomic data are available for *Leishmania* as well. Target-based drug development methods that block a single target molecule hardly ever produce the desired results, but bioinformatics approaches provided a ray of hope for this hypothesis (Schreiber et al., 2016).

### 10.1 Artificial intelligence to target autophagy in leishmaniasis

A potential field in systems biology, the use of artificial intelligence (AI) is receiving much attention as a result of the present data explosion. Over the decade, AI has revolutionized the process of drug discovery as a computational aspect. Autophagy has been studied using AI-based methods, as it has many other biological processes. He et al. (2020) used machine learning (ML) methods to categorize renal cell cancer (RCC) subtypes using autophagy proteins. Recently, various software that incorporate a wide range of ML algorithms have been created. For instance, Serrano et al. (2018) studied the impact of mRNA variations of a few autophagic genes, one pro-apoptotic gene and one anti-apoptotic gene in HIV-infected patients who were successfully treated with combined antiretroviral therapy using scikit-learn. Numerous applications of AI in drug discovery, including virtual screening and drug design, have been used (Walters and Barzilay, 2021).

Due to the growing accessibility of enormous pharmacological and chemical datasets, leishmaniasis and other NTDs (neglected tropical diseases) are being thoroughly investigated using ML models that use a non-clinical approach. Utilizing ML and virtual screening technologies, an organic compound dataset with activity against *L. amazonensis* was used to identify novel potential therapeutic options such as leishmanicides (Mollalo et al., 2018), (Tuon et al., 2022). Ornithine decarboxylase (ODC), a key enzyme in *Leishmania* proliferation, is inhibited by deguelin. Deguelin derivatives showed greater affinity and specificity against ODC than the original molecule using an *in silico*-generated combinatorial library and docking techniques. Thus, deguelin has been revealed as a promising approach for treating the infection by computer-aided drug design (CADD) (Vicente-Barrueco et al., 2022). The computational technique uses ML to build prediction models for molecular classification to find new leishmaniasis treatment components (Jamal and Scaria, 2013). Computational approaches also manifest crucial derivatives as a repurposing of the available drug to target specific processes. Since ATG8 of *Leishmania* plays a crucial role in infectivity and virulence (Giri and Shaha, 2019), it can be a potential target by using computational approaches for drug screening. Thiabendazole and urolithin A were shown to be possible drug molecules to combat against autophagy in leishmaniasis (Guhe et al., 2022), (Rahman et al, 2022.)

On the other hand, chemical agents that disrupt this pathway at the right molecule can be chosen if a crucial pathway is a target. Evidently, research on complex biological systems should acknowledge the constraints of simple systems in order to devise effective and safe therapeutics to treat disease. A systems biology approach, or a combination of experimental and computational study, is necessary to comprehend complex biological systems.

### 10.2 Systems biological approaches and its role in designing novel therapeutics

#### 10.2.1 Network systems biology

Cells can respond by changing their transcriptional activity, metabolism, or other regulatory processes in response to changes in their environment (internal stimuli and external stimuli) through signaling cascades. The role of these cells is crucial since it aids in cell survival and differentiation in various circumstances. It also controls the plasticity of cells in multicellular organisms. To understand the complex behavior of signaling networks, computational system modeling techniques were utilized to recreate conceptual models that highlight some fundamental characteristics of signaling routes and interpret prototypic signaling networks. Computational biology offers a solid platform to approach pertinent scientific issues through practical modeling and theoretical analysis.

Signaling pathways permit cells to perceive changes in their environment, external and internal stimuli, and respond by altering their transcriptional activity, metabolism, or other regulatory processes. Because it helps cell survival and differentiates in varied situations, the function of these cells is essential. Additionally, it controls the plasticity of cells in multicellular organisms. Computational systems modeling techniques were used to interpret prototypic signaling networks by reconstructing conceptual models that underline some key attributes of signaling pathways in order to comprehend the intricate behavior of signaling networks. Through pragmatic modeling and theoretical examination, computational biology provides a robust framework to address relevant scientific problems.

Network analysis in systems biology can be divided into two groups. Enrichment analysis falls under the first group. The objective of the second category, which is based on algorithm-related activities, is to identify possible targets or significant nodes in a network. It leads to centrality analysis, which has been used to identify the key nodes in the network. These techniques include radiality, clustering coefficient, betweenness centrality, and degree centrality. For the network-based study, the most widely used visualization and analysis software are Cytoscape (Shannon et al., 1971), Gephi (Bastian et al., 2009), Tulip (Auber et al., 2004), and Pajek (Mrvar and Batagelj, 2016). According to a bioinformatics study, NRF2 was interconnected to several signaling pathways while analyzing the complex autophagic network, including the AGE–RAGE pathway, MAPK pathway, NF-kappa B signaling, PI3K–Akt signaling, and VEGF signaling. This pathway enrichment was performed using “edgeR” R, and KEGG enrichment analysis was carried out with the DAVID system. Based on data analysis and experimental validation, NRF2 may be considered a tumor suppressor in tumorigenesis but promotes prostate cancer. Further confirmations offer more evidence that NRF2 is a crucial regulator and that blocking NRF2 and autophagy may be effective CRPC (castration-resistant prostate cancer) therapy (Zhang et al., 2022).

#### 10.2.2 Database availability for autophagy and *Leishmania*

When it comes to network analysis, systems biology studies mainly rely on biological databases. They provide access to a wide range of biologically significant data, such as the PPI (protein–protein interaction) network, disease–protein association data, microarray, next-generation sequencing, protein localization, post-translational modification, structural information about a protein or compound, and pathways related to proteins. However, there are no many databases that provide data about autophagy and about leishmaniasis. Databases available to the best of our knowledge based on host–pathogen interaction are enlisted in Table 3:

**TABLE 3:** Databases of autophagy and *Leishmania*.

Databases	Long form	URL	References
HAMDb	Human Autophagy Modulator Database	http://hamdb.scbdd.com	Wang et al. (2018)
ARN	Autophagy Regulatory Network	http://autophagyregulation.org/	Türei et al. (2015)
HADb	Human Autophagy Database	http://www.autophagy.lu/	Dr. Guy Berchem
ACDB	Autophagic Compound Database	http://www.acdbliulab.com	Deng et al. (2018)
Autophagy database	Autophagy database	http://tp-apg.genes.nig.ac.jp/autophagy	Homma et al. (2011)
AutophagySMDB	Autophagy Small Molecule Database	http://www.autophagysmdb.org	Nanduri et al. (2019)
LeishCyc	Biochemical pathway database for *L. major*	http://www.leishcyc.org	Doyle et al. (2009)
LmSmdB	An integrated database for metabolic and gene regulatory network in *L. major* and *Schistosoma mansoni*	http://www.nccs.res.in/LmSmdb/	Patel et al. (2016)

#### 10.2.3 Computational mathematical modeling in autophagy

A mathematical model serves as a reflector of the complexity of biological processes. A biological system’s complexity arises from a variety of causes. One of these is the system’s fundamental hierarchy, which extends from the level of cells to that of organisms. The hierarchical levels are all dynamic. Acute significant and stochastic alterations may occur at any time, even for minute changes in the cellular environment, even though they mimic regular and predictable activity. These uncertainties provide complications that are challenging for experimental research to fully understand. Even if they exist, it is difficult to understand the actual pathways and patterns of complex evolution. Because most autophagy biochemical events are non-linear, a small alteration in any one of its steps may not have a noticeable impact on the system as a whole.

To pinpoint the autophagy steps that oversee a specific system behavior, mathematical modeling supports reduced abstractions and approximations. Additionally, the system’s ongoing behavioral change introduces randomness into the autophagy process. In order to study the dynamics of the system in response to any changes in the environment brought on by numerous external or internal perturbations or signals, mathematical modeling of autophagy keeps track of these elements.

Experimental research and mathematical models have been used to demonstrate the intricate dynamics of autophagy. Studying the impact of starvation-induced autophagy on cell (yeast) population dynamics is a mathematical modeling application (Jin and Lei, 2014). Models investigate the dynamics of the proteins that control and influence various phases of macroautophagy. The fundamental set of proteins or pathways in various cellular conditions and diseases, such as cancer and Alzheimer’s disease, have been mathematically explored to capture the process of disease progression and to extract the significant parameter to restore cell homeostasis under disease situations (Han et al., 2012; Martin et al., 2013; Hao and Friedman, 2016; Enderling et al., 2007; Louzoun et al., 2014).

The computational model generated novel hypotheses that can be tested experimentally regarding the effects of ATG9 depletion and variation in LC3 copy number in mammalian cells (Martin et al., 2013). In conclusion, a mathematical model focuses on unraveling the intricate web of related autophagic processes in order to highlight the crucial ATG proteins or other molecules within the cascade. In processes like the destruction of pancreatic beta cells that cause diabetes or treatment of cancer, reactive oxygen species (ROS) dynamics are crucial. However, proposed data still fall short of a comprehensive explanation of existing data of cell metabolic and ROS dynamics. A computationally assisted mathematical model emphasized on the relationship between the metabolic processes in the cytoplasm, such as glycolysis, lactate and ATP production and consumption, and in the mitochondria, such as pyruvate reduction, NAD(P)H production, the function of the electron transport chain complexes, and others that control ROS dynamics (Fridlyand and Philipson, 2017).

By developing an ODE model, Kapuy et al. investigated Beclin1-mediated autophagy and caspase-mediated apoptosis. The B-cell lymphoma 2 (BCL2)–Beclin1–caspase minimal network was the focus of the model’s construction. The observation led to the hypothesis that the transition from autophagy to apoptosis is controlled by a bistable switch and that the sequential activation of cellular response can be started by a combination of BCL2-dependent regulation and feedback loops between Beclin1 and caspases depending on the intensity and duration of stress levels (Kapuy et al., 2013). Another study also suggested that TLR2/6 activates proinflammatory cytokines, whereas TNF-α activates PI3P to activate autophagy while causing autophagy inhibition by ATG9, showing the ATG9–PI3P axis acts as a negative feedback loop in autophagic machinery in the infection model of leishmaniasis (Guhe et al., 2022).

Although computational approaches have been studied in relation to other diseases, there are very few papers that highlight the autophagic behavior in leishmaniasis utilizing a systems biology approach, notably through mathematical modeling and network analysis. In order to help the researchers, the complexity of the autophagy network relevant to leishmaniasis is lessened, and this review recommends them to concentrate more of their efforts on computationally assisted techniques.

## 11 Synthetic biology

A fascinating field called synthetic biology emerged at the beginning of this century. It builds genetic programming systems for desired cellular behavior using molecular biology tools and advanced engineering ideas (Cameron et al., 2014). The goal is to replicate emergent biological processes using synthetic chemicals to develop artificial life. The other goals involve assembling systems with abnormal behavior using replaceable components from natural biology.

The term “synthetic biology” was introduced by Eric Kool and other speakers at the American Chemical Society’s annual meeting in San Francisco in 2000 (Benner and Sismour, 2005). Synthetic biology aims to expand the range of potential biological functions for therapeutic and scientific applications by making biology more efficient, dependable, and predictable to construct. By altering, recombining, and synthesizing wholly new genetic components, synthetic biologists rewire biological systems. This strategy is now feasible because there are more genetic building pieces available and we have a better grasp of how to assemble bimolecular modules, such as protein interaction networks and DNA regulatory sequences. The use of synthetic biology also benefits from developments in mathematical modeling and from engineering-related principles (Endy, 2005). Very few recent pieces of evidence are published that included autophagy modulation via genetic or synthetic circuits (Dey and Rangarajan, 2021), (Gupta et al., 2020). Literature also envisions the importance of the synthetic circuit in developing a leishmaniasis treatment plan (Mol et al., 2014), (Soni and Singh, 2022). There is still opportunity for research and the creation of new strategies for treating the disease by targeting the autophagic processes because, as far as we are aware, there are no studies that describe an autophagy regulation-based synthetic circuit in leishmaniasis.

## 12 Future perspective

A less explored and cardinal area in leishmanial infection is lipid metabolism, and it has become the focus nowadays showing its relationship with the autophagy process. The process of autophagy has been a key topic of research due to the intricate mechanism in many biological processes and diseases, because it is still a mystery with many unsolved issues, just like in leishmaniasis. The best methods to find these components might be systems biology-based ones. These methods can be used to gain a thorough understanding of the relationship and operation between lipids, miRNA, and autophagy. This study aims to highlight the interactions between essential biomolecules including RNA, proteins, and lipids that help maintain homeostasis and proper functioning of the cellular process of autophagy and also to identify novel strategies for the treatment of critical elements associated with these three key proteins for autophagy. Recent research on the function of autophagy in leishmaniasis and how it affects parasite survival has been widely disseminated, highlighting the growing significance of this process. The role of several biomolecules, including lipids, proteins, and mRNA, particularly miRNA, in modifying the autophagy process is another research focus. Recently, different ATG proteins such as LC3B, ATG9, ATG5, and ATG7 and other signaling molecules such as mTOR, FOXO, PI3K, and PDL have been shown to play a role in autophagy regulation in infection conditions. Study of autophagy in leishmaniasis is giving more attention to the PI3K/AKT pathway, PERK/ATF4 signaling, and FOXO1/SIRT1 axis. Moreover, LABCG2, an ABC family transporter; HSP90; ATG5; and an ATG8 homolog in *Leishmania* are a few more targets that have been found important for understanding leishmaniasis in experimental studies (Table 4).

**TABLE 4:** Probable targets in the host for regulating the autophagy process in leishmaniasis and its impact on the fate of parasite.

S.N.	Work showing impact on autophagy	Possible targets to combat against leishmaniasis	Reference
1	LAP decreases proliferation of parasites by inhibiting CD4^+^ T-cell growth	CD4^+^ Th1 cell-mediated immunity	(Okwor et al., 2014), (Peters et al., 2014), (Crauwels et al., 2015)
2	Protective Th1 response is restored when PDL-1 signaling is suppressed, which reduces the parasite load	PDL-1	Roy et al. (2019)
3	In BALB/c-infected mice, rapamycin or GSK-2126458 significantly reduced the parasite load	PI3K/AKT signaling	(Khadir et al., 2018), (Madeira Da Silva and Beverley, 2010), (Thomas et al., 2018)
4	*L. donovani* boosted AKT activation to control the GSK-3/-catenin/FOXO-1 axis which prevents apoptosis while contributing to parasite survival	GSK-3/-catenin/FOXO-1 axis	(Gupta et al., 2016)
5	SIRT1/FOXO-1 axis differentially regulates PD-1 signaling and intracellular parasite persistence	SIRT1/FOXO-1 axis	Roy et al. (2019)
6	ATG5 and ATG7 activate the PERK/ATF4 pathway, which favors cellular survival and parasite infection	PERK/ATF4 pathway	Zheng et al. (2019)
7	GSK2606414 binds to the kinase domain and shows anti-leishmanial activity	Kinase domain of PERK	Rangwala et al. (2014)
8	These miRNAs play a crucial role in autophagy of leishmaniasis	MicroRNAs MIR30A-3p, miR-101c, miR-129, and miR-210	(Singh et al., 2016), (Frank et al., 2015)

However, there is very little computational research compared to experimental studies. The review encourages filling these gaps to gain a better understanding of the dynamics of the autophagy pathways and their regulation in leishmaniasis. The literature has described the use of a variety of computational techniques in systems biology, including models based on mathematical modeling, AI-based approaches, synthetic biology-based techniques, genetic techniques, and image-based modeling, among many others. However, these techniques have not been sufficiently studied concerning leishmaniasis.

From the perspective of systems biology analysis, a handful of literature reported on autophagy under *Leishmania* infection. Such a study will help decipher the change in behavior of autophagy and its impact on parasites and help identify potential drug targets against disease. A structure-based study can be performed to determine the possible binding sites of the targets. The identification of binding sites of a protein can help in the rational designing of the therapeutic agents, which could be used to target processes by synthetic biology approaches via genetic circuits. This review also facilitates mathematical modeling to check the robustness of the network and for the identification of important nodes. A pipeline-based study is required in leishmaniasis for targeting the autophagic process and will surely aid to deliver fruitful insights into the role of autophagy in leishmaniasis and its impact on the proliferation and progression of the disease.

## 13 Concluding remarks

The need for new therapeutics against leishmaniasis is driven by the toxicity and drug resistance limitations of currently used chemotherapeutics. In leishmaniasis, it is unclear whether autophagy plays a role in disease progression or regression. Autophagy is a fundamental biological process that eliminates unwanted cellular components and pathogens in order to maintain cellular homeostasis. Due to its significant influence on cellular fate, autophagy is still dominating many cellular processes and signaling networks. Monitoring of genes involved in autophagy and signals that control autophagy have been used to identify and manage the autophagic pathways, and any disruption to any of them may result in several diseases. Given the complexity of autophagy and immunological signaling in disease systems and the growing attention to autophagy research in leishmaniasis, synthetic techniques may prove to be a successful therapeutic approach to cure leishmaniasis. Following many years of research, the experimental evidence on autophagy has grown, creating a thin-line intersection between lipids, proteins, and miRNA for the regulation of autophagy that needs to be investigated. Application of computational biology tools to these datasets may help in understanding information on the crucial proteins that regulate the autophagic processes and their underlying dynamics, which may significantly advance ongoing leishmaniasis research on its therapeutics. In conclusion, our study contributed to the advancement of autophagy research and showed how important biomolecules including lipids, proteins, and miRNA regulate this process. We advocate that in leishmaniasis, computational system biology tools may lead to a promising therapeutic approach for finding newer treatment regimens in the autophagic processes mediated by lipid, protein, and miRNAs.
